# Ghrelin Upregulates Oncogenic Aurora A to Promote Renal Cell Carcinoma Invasion

**DOI:** 10.3390/cancers11030303

**Published:** 2019-03-04

**Authors:** Tsung-Chieh Lin, Yuan-Ming Yeh, Wen-Lang Fan, Yu-Chan Chang, Wei-Ming Lin, Tse-Yen Yang, Michael Hsiao

**Affiliations:** 1 Genomic Medicine Core Laboratory, Chang Gung Memorial Hospital, Linkou 33305, Taiwan; tclin1980@gmail.com (T.-C.L.); yeh234@gmail.com (Y.-M.Y.); alangfan@gmail.com (W.-L.F.); 2 Genomics Research Center, Academia Sinica, Taipei 11529, Taiwan; jameskobe0@gmail.com; 3 Department of Diagnostic Radiology, Chang Gung Memorial Hospital, Chiayi Branch, Chang Gung University of Science and Technology, Chiayi 61363, Taiwan; weiming276@gmail.com; 4 Department of Medical Research, China Medical University Hospital, China Medical University, Taichung 40447, Taiwan; hardawayoung@gmail.com; 5 Department of Biochemistry, College of Medicine, Kaohsiung Medical University, Kaohsiung 80708, Taiwan

**Keywords:** ghrelin, aurora A, MMP10, invasion

## Abstract

Ghrelin is a peptide hormone, originally identified from the stomach, that functions as an endogenous ligand of the growth hormone secretagogue receptor (GHSR) and promotes growth hormone (GH) release and food intake. Increasing reports point out ghrelin’s role in cancer progression. We previously characterized ghrelin’s prognostic significance in the clear cell subtype of renal cell carcinoma (ccRCC), and its pro-metastatic ability via Snail-dependent cell migration. However, ghrelin’s activity in promoting cell invasion remains obscure. In this study, an Ingenuity Pathway Analysis (IPA)-based investigation of differentially expressed genes in Cancer Cell Line Encyclopedia (CCLE) dataset indicated the potential association of Aurora A with ghrelin in ccRCC metastasis. In addition, a significant correlation between ghrelin and Aurora A expression level in 15 ccRCC cell line was confirmed by variant probes. ccRCC patients with high ghrelin and Aurora A status were clinically associated with poor outcome. We further observed that ghrelin upregulated Aurora A at the protein and RNA levels and that ghrelin-induced ccRCC in vitro invasion and in vivo metastasis occurred in an Aurora A-dependent manner. Furthermore, MMP1, 2, 9 and 10 expressions are associated with poor outcome. In particular, MMP10 is significantly upregulated and required for the ghrelin-Aurora A axis to promote ccRCC invasion. The results of this study indicated a novel signaling mechanism in ccRCC metastasis.

## 1. Introduction

Cancer metastasis is one of the leading causes of cancer mortality. Approximately 30% of patients with renal cell carcinoma (RCC) present with metastatic disease [1]. Ghrelin, a peptide hormone, has been reported to promote cancer metastasis and is clinically associated with poor survival in various types of cancers [2]. However, ghrelin’s function in RCC remains largely unknown. We previously characterized its impact on cancer biology other than physiological role, and found that ghrelin increased clear cell type RCC (ccRCC) migration [3]. In ccRCC, immunohistochemical analysis of ghrelin indicated that ghrelin expression was increased in cancer tissues compared to normal adjacent tissues. In addition, ghrelin expression in RCC patients was associated with poor outcomes and with lymph node and distant metastasis. Furthermore, we found, for the first time, that ghrelin increased Snail protein levels and its promoter binding activity, leading to the E-cadherin downregulation, subsequently contributing to RCC migration [3]. Importantly, cancer metastasis is a complicated process with the involvement of multiple factors and genetic events which modulate several steps for initiating metastasis including tumor invasion at the primary site [4]. However, the mechanism of ghrelin-mediated RCC invasion has not yet been elucidated.

Aurora A (STK15/BTAK/hARK1/Aurora-2), a member of the serine/threonine Aurora kinase family, plays an important role in ensuring genetic stability in cell division. Aurora A is essential for mitotic spindle formation and accurate chromosome segregation [5]. Overexpression of Aurora A can induce centrosome amplification, aneuploidy and transformation of p53-deficient mammalian cells [6]. Recently, Aurora A was reported to associate with lymph node invasion in patients with breast cancer and renal cell carcinoma [7,8]. In an experimental metastasis model, breast cancer cells with Aurora A overexpression exhibited significant invasion to lung tissue in vivo [7]. Moreover, forced-expression of Aurora A increased the migration of laryngeal squamous cancer cells (LSCC), whereas stable knockdown of Aurora A inhibited cell migration in esophageal squamous cell carcinoma (ESCC) and breast cancer [7,9,10]. In addition, the pro-invasion function of Aurora A is likely to increase matrix metalloproteinase (MMP) expression in cancer cells [11]. These reports indicate a pivotal role and requirement of Aurora A in cancer cell invasion. However, the link between Aurora A and ccRCC metastasis and the signaling mechanism with regard to altered Aurora A function remains obscure.

The results from our clinical study indicate the correlation of the ghrelin-Aurora A axis with ccRCC invasion. To date, the issue of ghrelin-dependent regulation toward Aurora A in ccRCC has not been addressed. In this study, we aimed to investigate whether Aurora A is altered and required for ghrelin-induced ccRCC metastasis.

## 2. Results

### 2.1. The Analysis of the Cancer Cell Line Encyclopedia (CCLE) Dataset Via Ingenuity Pathway Analysis (IPA) Indicates that Aurora A Is Potentially Involved in Ghrelin-Mediated ccRCC Metastasis

**Figure 1:**
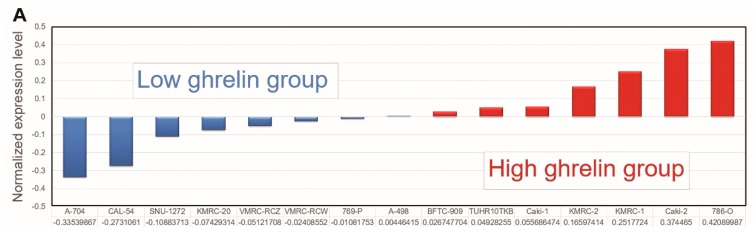

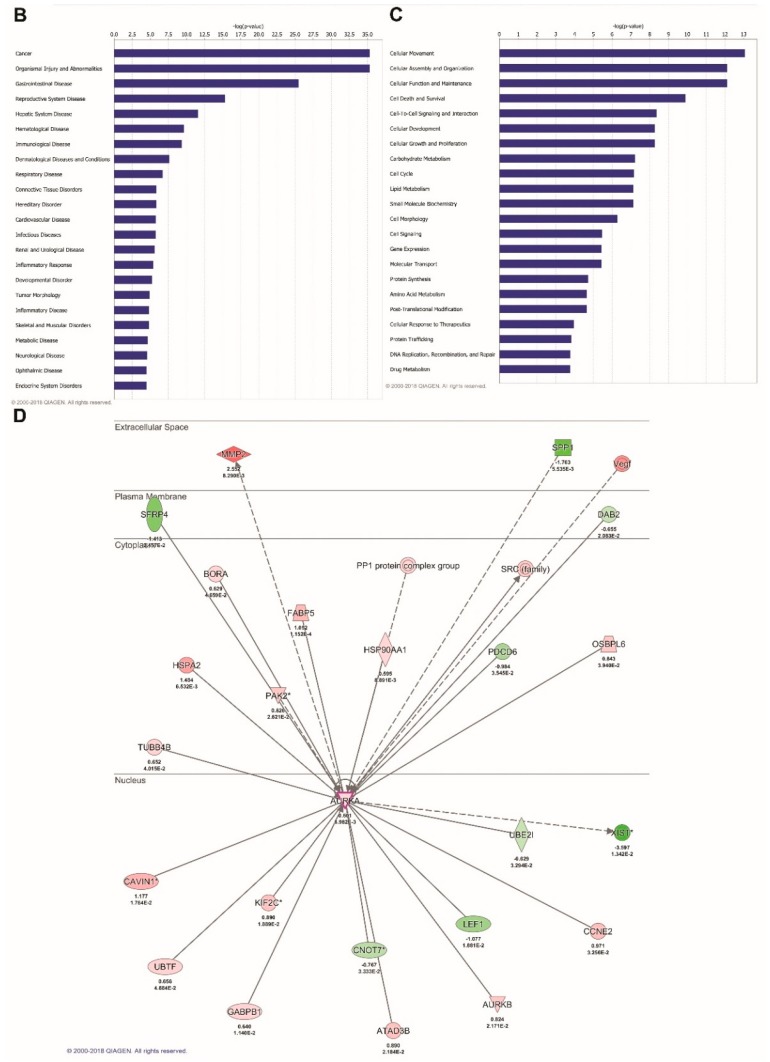
The analysis of Cancer Cell Line Encyclopedia (CCLE) dataset via Ingenuity Pathway Analysis (IPA) points out Aurora A is potentially involved in ghrelin-mediated ccRCC metastasis. (**A**) RCC cell lines and relative ghrelin expressions retrieved from CCLE dataset were listed. (**B**) 15 ccRCC cell lines were separated into high and low ghrelin groups. The differentially expressed genes between two groups were identified (threshold: >1.5 fold change and *p* < 0.05). Canonical pathway analysis was performed to rank the matched (**A**) Diseases and Disorders and (**C**) Molecular and Cellular Function in IPA database. (**D**) Statistical *P* values and log2 transformed expressions of Aurora A (*AURKA*) and its interactive network were shown. The red and green circles represent upregulation and downregulation, respectively.

We first comprehensively examined the impact of high ghrelin expression in ccRCC progression by a genome-wide analysis of differential gene expression in the Cancer Cell Line Encyclopedia (CCLE) [12,13]. 15 ccRCC cell lines were separated into high and low ghrelin groups based on the ranking determined by normalized expression level (GSE36133, Figure 1A). The differentially expressed genes between the two groups were selected (Table S1, threshold: > 1.5 fold change and *p* < 0.05) for further analysis using the IPA. 

Canonical pathway analysis was performed to identify Diseases and Disorders (Figure 1B) and Molecular and Cellular Function (Figure 1C) according to the matched differentially expressed genes in IPA database. The results revealed the association of high ghrelin expression with cancer and cellular movement (ranked top 1, Figure 1B,C). Furthermore, Aurora A was one of upregulated targets identified in high ghrelin group. The oncogenic role of Aurora A has been reported. However, little is known about its function in RCC progression. In addition, Aurora A’s interactive network had also been explored based on clinical data that MMP2 and VEGF were also increased in high ghrelin group (Figure 1D), indicating the potential value of studying ghrelin-Aurora A axis in RCC.

### 2.2. Aurora A Correlates with Poor Outcome in the ccRCC Cohort

**Figure 2:**
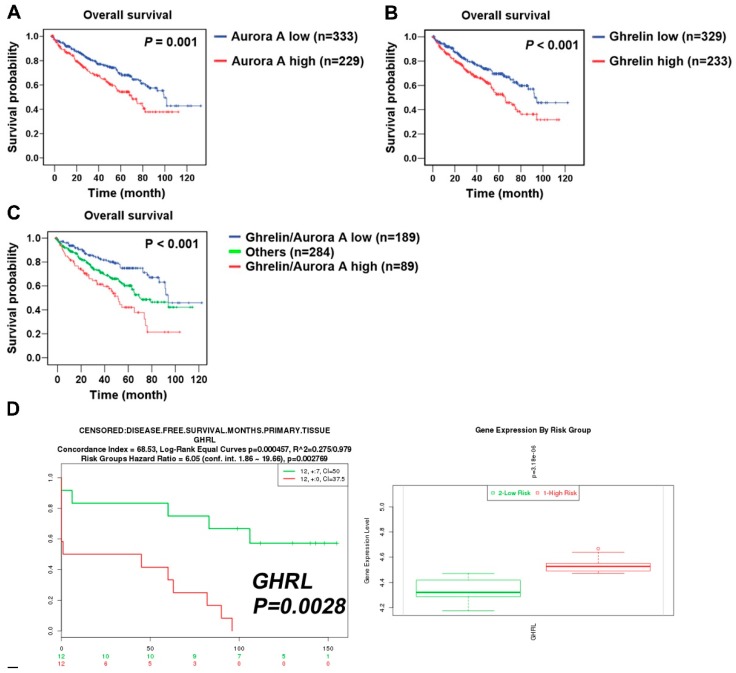

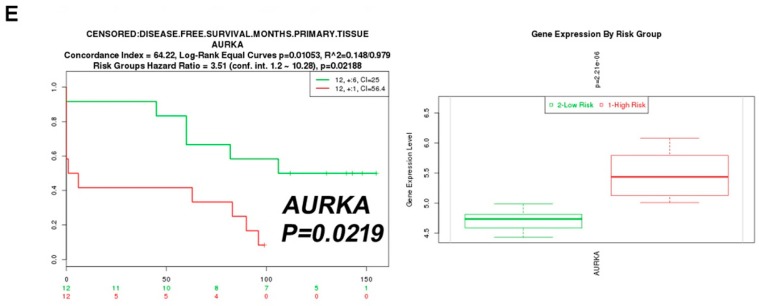
ccRCC patients with high ghrelin and Aurora A expression status correlates with poor outcome. Kaplan–Meier plot of cancer patients divided into high and low expression of Aurora A (**A**) and Ghrelin (**B**) were shown. (**C**) Kaplan–Meier plot of combining Aurora A and Ghrelin expression levels was analyzed. Data of 562 clear cell type RCC cases were retrieved from TCGA (KIRC gene expression (IlluminaHiSeq) dataset). The dataset showed the gene level transcription estimates, as in log2(x + 1) transformed RSEM normalized count. Subgroup was determined according to the ranking in expression level of indicated genes. (**D**) ccRCC patients of high GHRL (**D**) or AURKA (**E**) expression level correlates with poor disease-free survival. Data was analyzed using dataset (Wuttig Wirth Renal Kidney GSE22541), and was retrieved from the SurvExpress database.

To explore the clinical relevance of Aurora A expression in ccRCC patients, a cohort of 562 clear cell-type cases from The Cancer Genome Atlas (TCGA) was analyzed [14]. The Kaplan–Meier plot showed the correlation of high Aurora A expression with poor overall survival (*p* = 0.001, Figure 2A). We confirmed the previously identified ghrelin as a poor prognostic marker in the same cohort (Figure 2B). The combination of ghrelin with Aurora A status showed the prognostic power in predicting poor RCC survival (*p* < 0.001, Figure 2C). A disease-free survival analysis also revealed the association of ghrelin and Aurora A with poor outcome (Figure 2D,E). In addition, univariate and multivariate Cox regression analysis further indicated that high Aurora A level was a significant and independent predictor for high hazard ratios (Table 1). These results show the prognostic value of Aurora A for ccRCC patients.

### 2.3. Aurora A Expression Is Positively Associated with Ghrelin in ccRCC

**Figure 3:**
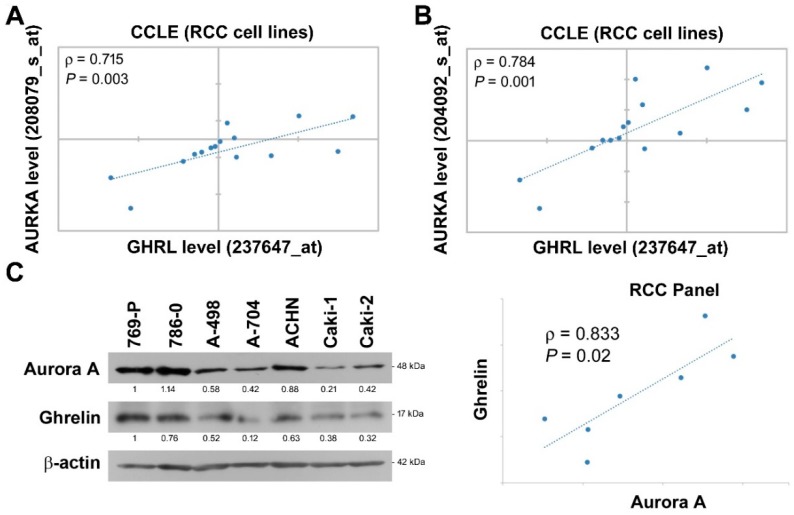
Ghrelin expression correlates with Aurora A in RCC cell lines. (**A**,**B**) Correlations in expression level of ghrelin and Aurora-A in ccRCC cell lines were respectively analyzed using different probes. Raw data was retrieved from CCLE dataset. (**C**) Endogenous Aurora A and ghrelin expression at protein level in ccRCC cell panel was determined by western blot method. Relative protein levels and statistical correlation were analyzed and shown after normalizing with β-actin internal control.

We further dissected the expressional correlation between Aurora A and ghrelin. Among ccRCC cell lines in CCLE dataset, the analysis was performed using variant Aurora A probes, and a positive correlation with *ρ* = 0.715 and 0.784 was observed respectively by each Aurora A probe (Figure 3A,B). In addition, endogenous ghrelin and Aurora A protein levels were explored in a panel of seven ccRCC cell lines (Figure 3C). The data further revealed a positive correlation of ghrelin and Aurora A at protein levels in ccRCC cell lines (*ρ* = 0.833).

### 2.4. Ghrelin Upregulates Aurora A

**Figure 4:**
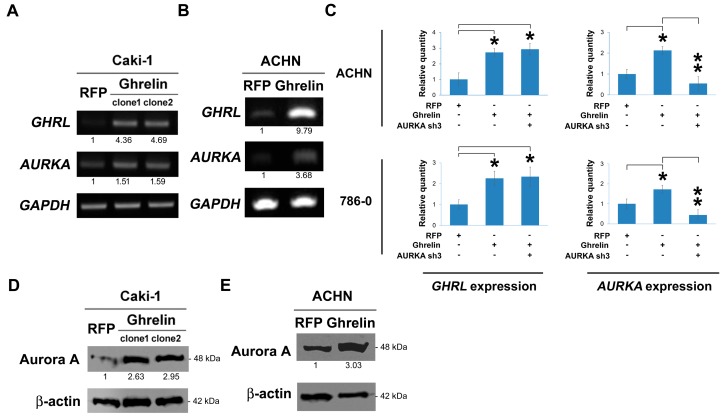
Ghrelin upregulates Aurora A in ccRCC. (**A**) RNA expression levels of indicated molecules were examined upon lentiviral-based ghrelin overexpression in Caki-1 cell clone 1 and clone2. (**B**) Regulation of ghrelin to Aurora A was investigated in ACHN cells using lentiviral-based overexpression method. (**C**) The modulation at RNA level was further examined by QPCR method. (**D**) The regulation of ghrelin overexpression to Aurora A protein was investigated by western blot in Caki-1 (**D**) and in ACHN cells (**E**). Figures were represented from the results of three repeated experiments with similar pattern. * *p* < 0.05, ** *p* < 0.01.

Ghrelin was stably overexpressed via lentiviral infection in Caki-1 cells. In the results, ghrelin ectopic overexpression elicited Aurora A upregulation at RNA level, respectively in clone 1 and clone 2 (Figure 4A) and ACHN cells (Figure 4B). A similar effect was observed by QPCR method (Figure 4C). In Caki-1 and ACHN cells, the increased Aurora A protein upon ghrelin overexpression was further observed and shown (Figure 4D,E). The data suggest a regulatory impact of ghrelin on Aurora A expression.

### 2.5. Aurora A Is Required for Ghrelin-Mediated ccRCC Invasion

**Figure 5:**
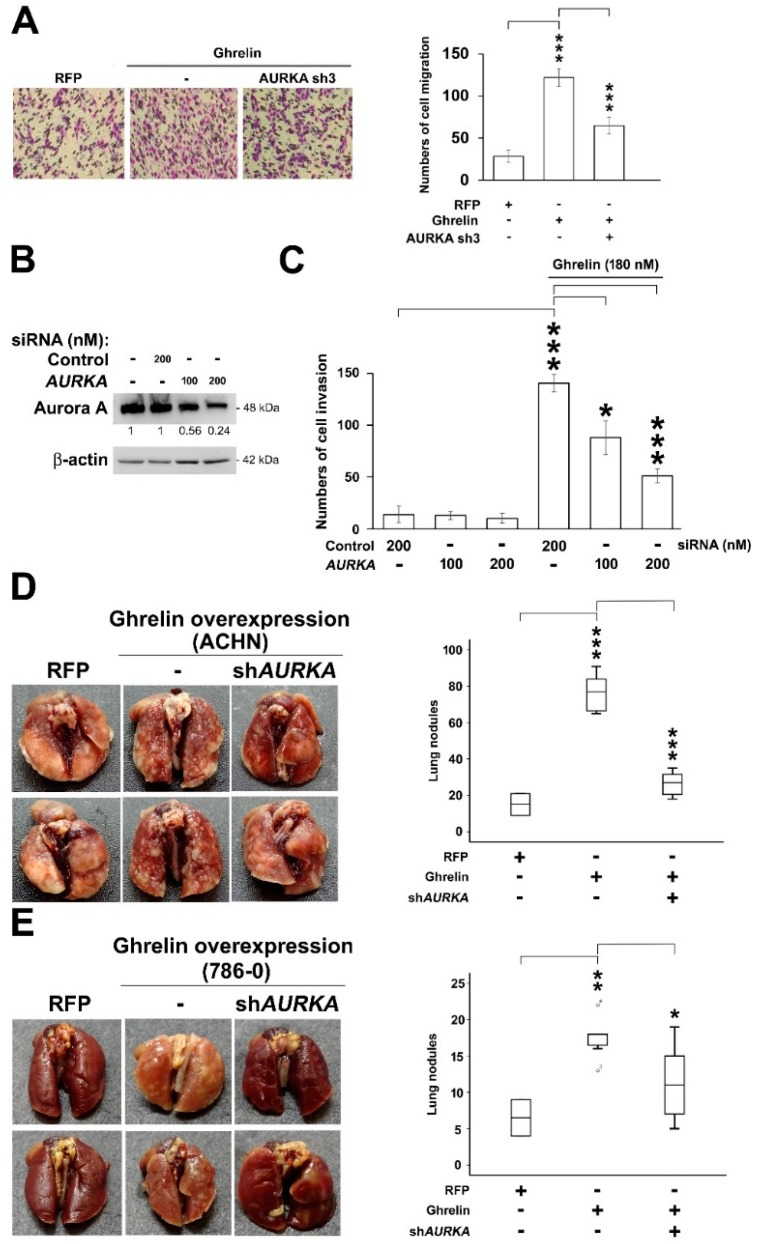
Aurora A is required in ghrelin-mediated RCC metastasis in vitro and in vivo. (**A**) Cell migration assay was performed by transwell devices using stable clones of ACHN cell. Numbers of cell migration in each group were counted after 5h of incubation. (**B**) Knockdown efficacy of Aurora A by specific siRNA in 786-0 cells was examined. Cells were treated with 100 or 200 nM of control siRNA or siRNA specific to Aurora A (**C**) Relative cell invasion ability in 786-0 cells upon Aurora A knockdown was studied. (**D**) RCC metastasis was investigated in ACHN cells (**D**) and in 786-0 cells (**E**) overexpressing ghrelin and combined stable Aurora A silencing. Representative images of lung surface nodule in indicated groups were showed (left). Numbers of lung nodule in each group were quantified 8 weeks after cell injection. *n* = 7 per each group (right). Figures were represented from the results of three repeated experiments with similar pattern. * *p* < 0.05, ** *p* < 0.01, *** *p* < 0.001.

Next, we aimed to explore whether ghrelin-induced ccRCC metastasis is dependent on Aurora A. Cell migration ability was first tested in ACHN cells, and the results showed the decrease in migrated cells upon Aurora A silencing in cells overexpressing ghrelin (*p* < 0.001, Figure 5A). Aurora-A was knocked down by 200 nM of specific siRNA in 786-0 cells which was the cell line characterized as having a high Aurora A background (Figure 5B). Aurora A silencing resulted in the reduction of cell invasion compared with the ghrelin treatment (Figure 5C). In in vivo metastasis model, Aurora A expression was stably reduced by shRNA in ACHN (Figure 5D) and 786-0 cells (Figure 5E) after ghrelin overexpression. Cells with Aurora A knockdown revealed the decreased lung metastasis as judged by lung nodules (right, Figure 5D,E). These data indicated the requirement of Aurora A in the ghrelin-mediated in vitro migration, invasion and in vivo metastasis in ccRCC.

### 2.6. MMP10 Is the Downstream Effector of the Ghrelin-Aurora A Signaling Axis in ccRCC Invasion

**Figure 6:**
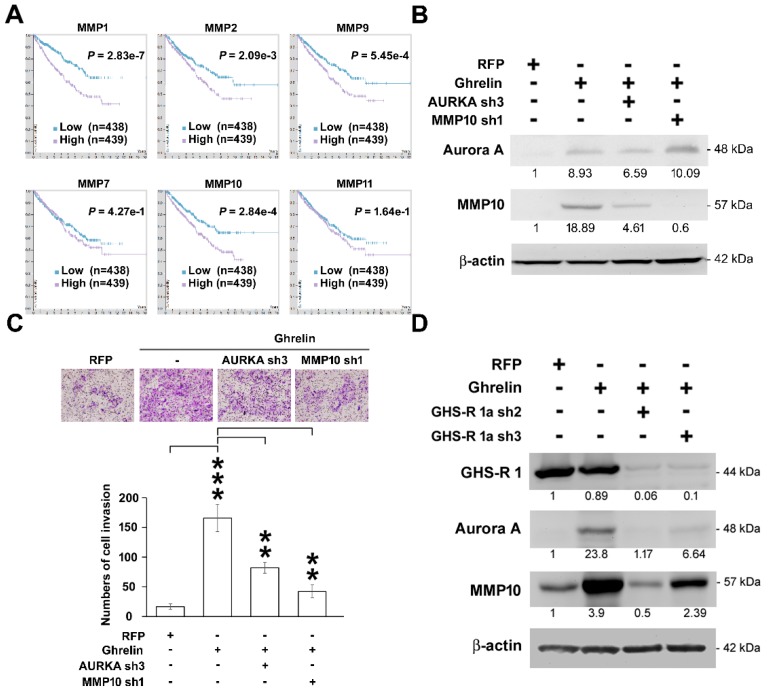
MMP10 is required in the ghrelin-Aurora A signaling axis to promote ccRCC invasion. (**A**) Kaplan–Meier plot showing the association of indicated MMP with RCC (TCGA) patient survival was represented. Data were retrieved from Human Protein Atlas website. (**B**) Representative Aurora A and MMP10 expression pattern in ACHN cells overexpressing ghrelin combined with Aurora A or MMP10 knockdown respectively. (**C**) Alternations in relative ACHN cell invasion ability were shown. Magnification: 100× (**D**) The regulation of ghrelin toward MMP10 expression was further studied in ACHN cells silencing GHS-R1a. Figures were represented from the results of three repeated experiments with similar pattern. ** *p* < 0.01, *** *p* < 0.001.

To study the potential regulation of ghrelin toward MMP expression that might be involved in the critical step for initiating cancer cell invasion, the association of MMPs including MMP1, 2, 7, 9, 10, 11 with cancer patient survival was explored to understand the correlation with clinical outcome. The data suggest a potentially pivotal role of indicated MMPs in the RCC invasion. In particular, the prognostic value of MMP10 in RCC was analyzed using The Human Protein Atlas database, which verified the consequences of transcript levels linking to patient survival outcomes [15,16,17,18,19]. The high level of MMP10 in renal cancer patients was found to be associated with poor survival (*p* = 0.000284, Figure 6A). In addition, MMP10 upregulation was reduced after Aurora A silencing in ACHN cells (Figure 6B). The impact of MMP10 alternation was examined in cell invasion test, which showed the decrease in invasive cell numbers upon Aurora A or MMP10 silencing (Figure 6C). The ghrelin receptor, GHS-R1a, was relatively silenced by specific shRNAs (clone sh2 and sh3), and knockdown of GHS-R1a blocked the signaling axis elicited by ghrelin overexpression (Figure 6D). The result indicated the increase in MMP10 level contributed to ccRCC invasion ability, and characterized the importance of the ghrelin-ghrelin receptor-Aurora A-MMP10 signaling pathway in ccRCC metastasis.

## 3. Discussion

Renal cell carcinoma (RCC), also called renal adenocarcinoma, comprises 90–95% of kidney-derived tumors, and is a form of kidney cancer that arises from the cells of the renal tubule [20]. Although RCC is relatively rare compared with other cancers (approximately 2% of malignant tumors), an alarming increase in incidence has been diagnosed and the survival of these patients is poor, with a median survival of less than one year [21]. In addition, about 30% of RCC patients present with metastatic disease, the metastatic RCC (mRCC). The common sites of metastasis include lung, lymph node, bone and brain [1]. In particular, mRCC is generally resistant to chemotherapy. Immunologic therapy with interferon or interleukin-2 (IL-2) has been the most commonly used treatment, despite low a response rate (5–20%) [1]. Hence, it is urgently required to unravel the molecular mechanisms involved in tumorigenesis and metastasis of mRCC for the development of novel target agents. We previously identified a peptide hormone, ghrelin, and investigated the function and mechanism of ghrelin in RCC metastasis [3]. The result of immunohistochemical analysis of ghrelin showed an increase in ghrelin expression in specimens obtained from individuals with disease progression and a progressive ghrelin upregulation in cancer tissues compared to normal adjacent tissues. Furthermore, ghrelin expression is correlated with poor outcome, lymph node and distant metastasis status in RCC patients. Our previous studies indicated that ghrelin could increase Snail protein level and its E-cadherin promoter binding activity via phosphatidylinositol 3-kinase–Akt signaling activation, leading to downregulated E-cadherin expression and subsequently contributing to the development of EMT and RCC migration. The study demonstrated the poor prognostic and pro-metastatic role of ghrelin in RCC. Importantly, cancer metastasis is a complicated process that requires multiple factors to elicit tumor invasion at the primary site. We first observed that MMP10 is increased upon ghrelin treatment in clear cell type of RCC, suggesting a novel function of ghrelin in promoting RCC metastasis.

In this study, we observed Aurora A upregulation by ghrelin, especially at the RNA level, suggesting a potential transcriptional activation of the *AURKA* gene. Recently, increasing reports point out that Aurora A is a target of Wnt/β-catenin signaling pathway, which is involved in multiple myeloma disease progression [22]. In particular, *AURKA* expression is driven by β-catenin transcription in VHL-null ccRCC. However, whether Wnt/β-catenin signaling is activated by ghrelin leading *AURKA* transcriptional activation remains to be explored. An investigation of the synergistic effect of hepatocyte growth factor (HGF) and vascular endothelial growth factor (VEGF) in human endothelial cells revealed the increased expression of human *AURKA* mRNA in cultured cells 24 hours after initial treatment [23]. Furthermore, co-expression of GABPA and GABPB1 proteins significantly increased the promoter (-189-354) activity of human *AURKA* gene [24]. Interestingly, both VEGF and GABPB1 were found to be upregulated in high ghrelin group in the CCLE dataset (Figure 1D), indicating the regulatory mechanism of oncogenic Aurora A upregulation that remains to be studied. In addition, *BORA* is a known Aurora A cofactor required for its kinase activity [25]. The BORA level was also increased in high ghrelin group suggesting a potential role of ghrelin in activating Aurora A in RCC. A report indicated that YY1 could suppress invasion and metastasis by downregulating MMP10 in a MUC4/ErbB2/p38/MEF2C-dependent manner in pancreatic cancer cells, suggesting MEF2C phosphorylation is required for MMP10 expression [26]. Thus far, the link of ghrelin and Aurora kinase A to MEF2C phosphorylation has not yet been studied, and this link might shed light on the molecular mechanism of MMP10 upregulation during RCC metastasis.

Similar function of ghrelin was indicated in gastric cancer invasion of which mechanism was unraveled, that is, via the activation of GHS-R/NFκB signaling pathway [27]. In addition to the modulation of cancer invasion, the ghrelin-ghrelin receptor signaling axis is pivotal in regulating cell motility and cell-cell adhesion, which led to cancer metastasis in many types of cancer [2]. Ghrelin treatment could activate PI3K/GTP-Rac signaling resulting in the actin polymerization in astrocytoma cells [28]. According to the results of a pancreatic adenocarcinoma study, ghrelin promoted cell migration via the activation of GHSR/PI3K/Akt signaling pathway, and the phenotype was inhibited by the addition of ghrelin receptor antagonist [29]. A study in colorectal cancer revealed that the pretreatment of antagonist D(Lys-3)-GHRP-6 inhibited ghrelin-mediated ghrelin receptor function and cell migration ability [30]. Moreover, ghrelin was also observed to induce cell migration by triggering the activation of GHSR/CaMKII/AMPK/NFκB signaling pathway in glioma cells [31]. In our previous study, ghrelin was found to reduce cell-cell contact in cell migration process through Snail-dependent E-cadherin repression. Taken together, the findings demonstrate ghrelin’s multi-function in promoting cancer metastasis.

## 4. Materials and Methods

### 4.1. Ingenuity Pathway Analysis (IPA)

Differential gene expression signatures of ccRCC cohort divided into high ghrelin and low ghrelin groups were analyzed by Ingenuity^®^ Pathway Analysis (QIAGEN, Hilden, Germany; www.qiagen.com/ingenuity), according to the instructions provided. After comparison of the imported dataset with Ingenuity^®^ Knowledge Base, a list of relevant networks, upstream regulators and algorithmically generated mechanistic networks based on the connectivity was obtained. The Canonical Pathway analysis of IPA was used to rank significant Diseases and Disorders, Molecular and Cellular Functions based on the altered gene signatures.

### 4.2. Cell Culture

Human renal adenocarcinoma cell lines were all obtained from American Type Culture Collection (Manassas, VA, USA). 786-0 cells were maintained in RPMI 1640 medium supplemented with 10% fetal bovine serum (GIBCO, Grand Island, NY, USA), 10 mM HEPES, 1 mM sodium pyruvate, penicillin (100 unit/mL), and streptomycin (100 μg/mL). 769-P cells were maintained in RPMI 1640 medium supplemented with 10% fetal bovine serum (GIBCO, Grand Island, NY, USA), penicillin (100 unit/mL), and streptomycin (100 μg/mL). ACHN, A-498 and A-704 cells were maintained in MEM medium supplemented with 10% fetal bovine serum, penicillin (100 unit/mL), and streptomycin (100 μg/mL). Caki-1 and Caki-2 cells were maintained in McCoy’s 5a medium supplemented with 10% fetal bovine serum, penicillin (100 unit/mL), and streptomycin (100 μg/mL). Cells were incubated in 95% air, 5% CO_2_ humidified atmosphere at 37 °C. Ghrelin (n-octanoyl) was obtained from ANASPEC (Fremont, CA, USA). Acylated ghrelin (*n*-octanoyl) was prepared in ddH_2_O.

### 4.3. Preparation of Ghrelin Expression Plasmid

Ghrelin was cloned from 293T cDNA using TAKARA DNA polymerase (Mountain View, CA, USA) according to the manufacture’s instruction. The primer sequences designed were as follows: ACCCAAGCTGGCTAGCATGCCCTCCCCAGGGACCGTC (sense) and TCAAGAT CTAGAATTCTCACTTGTCGGCTGGGGCCTC (antisense). The PCR products were gel-purified, digested with NheI/EcoRI, and subcloned into lentiviral expression vector pLAS3W (RNAi Core, Academia Sinica, Taipei, Taiwan). The sequences were confirmed via DNA sequencing by Sequencing Core Facility, SIC, Academia Sinica.

### 4.4. Animal Study

All animal experiments were conducted in accordance with a protocol approved by the Academia Sinica Institutional Animal Care and Utilization Committee (ethical code: 12-02-319, 18 October 2016). Age-matched male NSG mice (6 to 8 weeks of age) were used. To evaluate metastasis, 1 × 10^6^ cells were resuspended in 0.1 mL of PBS and injected into the lateral tail vein (*n* = 7). Metastatic lung nodules were counted and were further confirmed via HE staining using a dissecting microscope (OLYMPUS, Tokyo, Japan).

### 4.5. Lentivirus-Based shRNA Production and Infection

The lentiviral shRNA constructs were purchased from Thermo Scientific (Pittsburgh, PA, USA). Lentiviruses were produced via co-transfection of 293T cells with an shRNA-expressing plasmid, an envelope plasmid (pMD.G) and a packaging plasmid (pCMV-dR8.91) using calcium phosphate (Invitrogen, Carlsbad, CA, USA). The 293T cells were incubated for 18 h, followed by replacement of the culture medium. The viral supernatants were harvested and titered at 48 and 72 h post-transfection. The cell monolayers were infected with the indicated lentivirus in the presence of polybrene and were further selected using puromycin (4 μg/mL) for 7 days. The selected stable clones were further cultured in the presence of 2 μg/mL puromycin.

### 4.6. Western Blot Analysis

The cells were lysed at 4 °C in RIPA buffer containing 50 mM Tris-HCl (pH 7.4), 150 mM NaCl, 1% Triton X-100, 0.25% sodium deoxycholate, 5 mM EDTA (pH 8.0), and 1 mM EGTA supplemented with protease and phosphatase inhibitors. After 20 min of lysis on ice, the cell debris was removed via microcentrifugation, followed by rapid freezing of the supernatants. The protein concentration was determined using the Bradford method. In our experiments, equivalent loads of 25–50 μg of protein were electrophoresed using a SDS-polyacrylamide gel and then electrophoretically transferred from the gel to a PVDF membrane (Millipore, Bedford, MA, USA). After blocking with 5% non-fat milk, the membrane was incubated in specific primary antibodies (Ghrelin: GTX10473, GeneTex, Irvine city, CA, USA, 1:1000; Aurora A: #4718, Cell Signaling, Danvers, MA, USA, 1:1000; MMP10: sc-80197, Santa Cruz, Dallas, TX, USA, 1:1000; β-actin: A5316, Sigma-Aldrich, Louis, MO, USA, 1:5000; GHS-R 1: sc-374515, Santa Cruz, 1:2000) overnight at 4 °C and subsequently incubated in a corresponding horseradish peroxidase-conjugated secondary antibody for 1 h. The membranes were visualized using the ECL-Plus detection kit (PerkinElmer Life Sciences, Boston, MA, USA).

### 4.7. Invasion and Migration Assay

The in vitro migration and invasion were assessed using Transwell assay (Millipore, Bedford, MA, USA). For invasion assay, transwell was additional pre-coated with 35 μL of 3× diluted matrix matrigel (BD Biosciences Pharmingen, San Diego, CA, USA) for 30 min. Cells of 2 × 10^5^ in serum-free culture medium were added to the upper chamber of the device, and the lower chamber was filled with 10% FBS culture medium. After indicated hours of incubation, upper surface of the filter was carefully removed with a cotton swab. The filter was then fixed, stained and photographed. Cells invasion was quantified by counting the cells in three random fields per filter.

### 4.8. Semi-Quantitative RT-PCR and Real-Time PCR Amplification Analysis 

Total cellular RNA was extracted by TRIzol reagent (Invitrogen, Carlsbad, CA, USA) in accordance with the manufacturer’s instructions. One microgram of total RNA was reverse-transcribed using Advantage RT for PCR Kit (Clontech, Mountain View, CA, USA) at 42 °C for 1 h as described in the manufacturer’s protocol. PCR conditions for rat leptin were 94 °C for 5 min and 37 cycles at 94 °C for 30 s, 56 °C for 30 s and 72 °C for 60 s, followed by a final extension step at 72 °C for 5 min by Bio-Rad icycler (Bio-Rad, Oxford, UK). For each combination of primers, the kinetics of PCR amplification was studied. The number of cycles corresponding to plateau was determined and PCR was performed at exponential range. PCR products were then electrophoresed through a 1% agarose gel and visualized by ethidium bromide staining in UV irradiation. The mRNA levels were also determined by real-time PCR with ABI StepOnePlus real-time PCR system according to the manufacturer’s instructions. GAPDH was used as endogenous control. PCR reaction mixture contained the SYBR PCR master mix, 50 ng cDNA, and primers. Relative gene expression level that the amount of target were normalized to endogenous control gene was calculated using the comparative Ct method formula E^−ΔΔCt^. The relative primer sequences for semi-qPCR are listed below: GHRL_F: 5′-GAGCCCTGAAC ACCAGAGAG-3′, GHRL_R: 5′-CCCAGAGGATGTCCTGAAGA-3′ (239 bp); AURKA_F: 5′-TGG AATATGCACCACTTGGA-3′, AURKA_R: 5′-ACTGACCACCCAAAAT CTGC-3′ (208 bp); GAPDH_F: 5′-GCTGAGAACGGGAAGCTTGT-3′, GAPDH_R: 5′-GCCAGGGGTGCTAAGCA GTT-3′ (299 bp). The relative primer sequences for real-time PCR are listed below: GHRL_F: 5′-GGCATCTGACCTCCACTGTT-3′, GHRL_R: 5′-TCTAAACCAGCAACC CCATC-3′ (119 bp); AURKA_F: 5′-TTGGAAGACTTGGGTCCTTG-3′, AURKA_R: 5′-ACGTTTTGGACCTCCAA CTG-3′ (119 bp); GAPDH_F: 5′-GACAGTCAGCCGCATCTTCT-3′, GAPDH_R: 5′-GCGCCCAA TACGACCAAATC-3′ (104 bp).

### 4.9. Statistical Analysis

Estimates of the survival rates were calculated using the Kaplan-Meier method and were compared using the log-rank test. The association between clinicopathological categorical variables and *AURKA* expression was analyzed using the chi-squared test. Student’s *t*-test was used for other statistical analyses. All data are presented as the mean ± S.D. The *p* values at the following levels were considered to be significant: * *p* < 0.05, ** *p* < 0.01, and *** *p* < 0.001. All data was represented based on three repeated experiments with similar pattern.

## 5. Conclusions

In summary, the analytical results from the CCLE database revealed a significant association between ghrelin and Aurora A expression in ccRCC. In addition, patients with high ghrelin and Aurora A status have poor outcomes. We further observed that ghrelin could upregulate Aurora A at the protein and RNA levels and that Aurora A plays a pivotal role in ghrelin-induced RCC invasion and in vivo metastasis. Among those MMPs identified, MMP10 was associated with poor survival in ccRCC, and the upregulation of MMP10 was induced by the ghrelin-ghrelin receptor-Aurora A signaling axis to promote ccRCC metastasis.

## Figures and Tables

**Table 1:** Cox univariate and multivariate regression analysis of TNM prognostic factors and Aurora A expression for overall survival in 562 renal cell carcinoma patients.

Variable	Comparison	Univariate		Multivariate	
		HR (95% CI)	*p*	HR (95% CI)	*p*
T	T1-T2; T3-T4	3.422 (2.488–4.708)	<0.001	2.555 (1.599–4.083)	<0.001
N	N0; N1-N3	2.618 (1.39–4.929)	0.003	1.356 (0.698–2.632)	0.369
M	M0; M1	4.429 (3.208–6.115)	<0.001	2.707 (1.665–4.399)	<0.001
Aurora A	Low; High	1.731 (1.268–2.363)	0.001	1.645 (1.086–2.491)	0.019

Note: Cox proportional hazards regression was used to test independent prognostic contribution of Aurora A after accounting of other potentially important covariates. Abbreviation: HR, hazard ratio; CI: confidence interval.

## Supplementary Materials

The following are available online at https://www.mdpi.com/2072-6694/11/3/303/s1, Table S1: Supplementary data.
